# Medical Expenditures Associated With Diabetes Among Adult Medicaid Enrollees in Eight States

**DOI:** 10.5888/pcd15.180148

**Published:** 2018-09-27

**Authors:** Boon Peng Ng, Sundar S. Shrestha, Andrew Lanza, Bryce Smith, Ping Zhang

**Affiliations:** 1 Division of Diabetes Translation, Centers for Disease Control and Prevention, Atlanta, Georgia

## Abstract

**Introduction:**

Little information is available on state-specific financial burdens of diabetes in the Medicaid population, yet such information is essential for state Medicaid programs to plan diabetes care and evaluate the benefits of diabetes prevention. We estimated medical expenditures associated with diabetes among adult Medicaid enrollees in 8 states.

**Methods:**

We analyzed the latest available 2012 CMS Medicaid claims data for 1,193,811 adult enrollees aged 19–64 years in 8 states: Alabama, California, Connecticut, Florida, Illinois, Iowa, New York, and Oklahoma. For each state, we stratified the study population by Medicaid eligibility criteria: disability and nondisability. For each group, we estimated per capita annual medical expenditures on outpatient care, inpatient care, and prescription drugs by using a 2-part model, adjusted for age, sex, race/ethnicity, and comorbidities. We calculated the expenditures associated with diabetes as the difference in predicted expenditures for enrollees with and without diabetes. Analyses were done in 2017.

**Results:**

For disability-based enrollees, the estimated total per capita annual diabetes expenditures ranged from $6,183 in Alabama to $15,319 in New York (all *P* < .001). For nondisability-based enrollees, the corresponding estimates ranged from $4,985 in Alabama to $15,366 in New York (all *P* < .001). The proportion of individual components varied by state and eligibility criteria.

**Conclusion:**

Medical expenditures associated with diabetes among adults on Medicaid were substantial and varied across studied states. Our estimates can be used by the 8 state Medicaid programs to prepare health care resources needed for diabetes care and assess the financial benefits of diabetes prevention programs.

## Introduction

As a program jointly funded by the federal government and the states, Medicaid plays an important role in providing health care coverage for adults (about 12.9 million low-income adults and 9.8 million disabled adults in 2012) (1). Medicaid is especially important for those with diabetes, as the disease affects low-income individuals disproportionately, and individuals living in poverty are more likely to develop diabetes-related complications (2,3). In 2012, about 14% of adults aged under 65 years, covered by Medicaid, had diabetes (4).

The financial burden imposed on Medicaid programs by diabetes is substantial; in 2013, medical expenditures associated with diabetes paid by Medicaid programs was estimated to be $25.7 billion (5). Medical spending on the Medicaid population is expected to rise in the future because of increases in 1) the number of people with diabetes enrolled in Medicaid programs, resulting from the growing prevalence of people with diabetes (6), expansion of Medicaid programs (7), or both, and 2) per capita medical expenditures associated with diabetes. Per capita medical spending associated with diabetes has been increasing over time (8), and the trend is expected to continue in the foreseeable future (9).

Prior studies on medical expenditures associated with diabetes mainly focused on national-level estimates (3,7). Using data from the Medical Expenditure Panel Survey, Garfield and colleagues calculated that in 2008 nationally, Medicaid spent an average of $9,401 more (more than 3 times higher) on adult enrollees with diabetes than those without diabetes (7). However, as Medicaid eligibility criteria and coverage policies vary greatly by state, these estimates do not reflect expenditures at the state level. The Kaiser Family Foundation estimated that, in 2011, the average annual medical spending per Medicaid enrollee varied from a low of $4,010 in Nevada to a high of $11,091 in Massachusetts (10). In addition, previously reported national estimates did not consider variations in medical expenditures by age group and Medicaid eligibility criteria. Based on the same study by the Kaiser Family Foundation, per capita annual medical spending for children and nondisabled adults was substantially lower ($2,492 and $4,141, respectively) than for older adults and disabled adults ($17,522 and $18,518, respectively) (10).

To plan heath care resources needed for diabetes care and assess the financial benefits of prevention and management programs, decision makers at state Medicaid programs need estimates that reflect the actual expenditure in their states. To that end, we estimated the medical expenditures associated with diabetes among Medicaid adult enrollees aged 19–64 years by disability-based eligibility status (ie, disabled or nondisabled) in 8 states using state-specific data.

## Methods

### Data

We analyzed Medicaid administrative claims data, also known as Medicaid Analytic eXtract (MAX) files, from the Chronic Conditions Data Warehouse (CCW) of the Centers for Medicare and Medicaid Services (CMS), which includes 100% of fee-for-service (FFS) enrollees. The MAX files were compiled from the Medicaid Statistical Information System at the state level. The files contain annual Medicaid enrollment information, medical utilization claims, and expenditure of services for Medicaid enrollees at the individual level in all 50 states and the District of Columbia (11). All health care service claims, expenditures, and enrollment information were linked through encrypted beneficiary identifiers (11).

### Study population

Our study population included 1,193,811 adult Medicaid enrollees aged 19–64 years who were enrolled in an FFS plan for the whole calendar year 2012, the latest available data at the time of the study. For each state, we created 2 subgroups based on enrollees’ disability or nondisability status, using the basis of eligibility indicators in the MAX personal summary file. In each group, enrollees were identified with diagnosed diabetes if they had at least 1 inpatient or 2 outpatient claims 30 days apart based on a primary or secondary diagnosis of the International Classification of Diseases, Ninth Revision, Clinical Modification (ICD-9-CM) codes for diabetes (250x, 357.2x, 362.0x, 366.41) (12).

The following enrollees were excluded.
  - Those with restricted benefits, such as benefits for pregnancy-related services only, for family planning services only, and for benefits based on dual eligibility status (eligible for both Medicare and Medicaid) (13).
  - Those without demographic information (age, sex, race/ethnicity).
  - Pregnant women, because they have different medical or treatment needs.
  - Those in long-term care facilities, because diagnosis codes reflected patients’ diagnoses at admission to the facility. Their health care utilizations are therefore not fully captured (11).
  - States with data anomalies, such as states that did not provide data as required by CMS (13).

### Selection of states

To ensure a reliable estimate for each of the medical expenditure components (inpatient care, outpatient care, and prescription drug expenditures), we set a minimum analytical sample size of 1,000 for each of the study groups, based on Medicaid eligibility criteria and diabetes status. There were 2 eligibility criteria for Medicaid enrollees: disability-based and nondisability-based. Combining these eligibility criteria with diabetes status yielded 4 study groups: people with diabetes and disability, people without diabetes but with disability, people with diabetes but without disability, and people without diabetes and without disability. Only 7 states (Alabama, California, Connecticut, Florida, Illinois, New York, and Oklahoma) had analytical sample sizes of more than 1,000 enrollees for all 4 groups. In addition, we included Iowa although the nondisability and diabetes group had only 935 enrollees (Table 1).

**Table 1:** Characteristics and Unadjusted Total Medical Expenditures of Adult Medicaid Enrollees Aged 19–64 Years by Disability-Based Eligibility and Diabetes Status, 8 States, 2012

State and Variable	With disability		*P* value^a^	Without disability		*P* value^a^
	With diabetes	Without diabetes		With diabetes	Without diabetes	
**Alabama**						
Sample size, N	11,784	52,461	–	1,365	17,137	–
Mean age, y	51.1	42.4	<.01	39.2	32.8	<.01
Female, %	70.6	53.3	<.01	89.7	88.1	.08
**Race/ethnicity, %**						
White	45.6	48.1	<.01	42.9	48.4	<.01
Black	53.4	50.8	<.01	55.7	50.0	<.01
Hispanic	0.5	0.5	.97	1.0	0.9	.68
Other	0.5	0.6	.11	0.4	0.6	.42
**Comorbidity^b^, % **						
Dementia	0.4	0.2	<.01	0.1	0.1	.83
Chronic pulmonary disease	32.0	21.0	<.01	23.9	15.6	<.01
Rheumatic disease	2.6	2.2	<.01	2.9	1.4	<.01
Peptic ulcer disease	1.7	1.0	<.01	1.6	0.9	.01
Hemiplegia	0.6	0.8	.06	0.3	0.1	.03
Any malignancy	8.8	4.6	<.01	7.5	4.2	<.01
Mild liver disease	1.7	1.0	<.01	0.4	0.2	.01
Moderate/severe liver disease	0.7	0.4	< .01	0.4	0.2	.01
Metastatic tumor	0.7	0.6	.67	1.2	0.6	<.01
AIDS	0.8	1.4	<.001	0.5	0.4	.49
Per capita medical expenditures^c^, $	13,424	7,794	<.01	8,069	3,747	<.01
**California**						
Sample, N	5,619	44,703	–	3,505	99,848	–
Mean age, y	53.1	42.2	<.01	46.5	33.9	<.01
Female, %	60.2	49.1	<.01	67.8	63.9	<.01
**Race/ethnicity, %**						
White	61.8	66.6	<.01	31.8	42.1	<.01
Black	5.2	8.8	<.01	3.6	6.0	<.01
Hispanic	22.6	16.4	<.01	52.4	39.6	<.01
Other	10.4	8.2	<.01	12.2	12.2	.95
**Comorbidity^b^, %**						
Dementia	0.3	0.2	<.01	0.0	0.0	.65
Chronic pulmonary disease	28.5	15.2	<.01	12.1	6.2	<.01
Rheumatic disease	3.0	1.8	<.01	1.7	0.5	<.01
Peptic ulcer disease	1.6	0.7	<.01	0.6	0.3	<.01
Hemiplegia	1.1	1.0	.23	0.3	0.1	<.01
Any malignancy	6.9	4.0	<.01	15.6	3.8	<.01
Mild liver disease	4.0	1.7	<.01	1.1	0.2	<.01
Moderate/severe liver disease	2.3	0.8	<.01	0.5	0.1	<.01
Metastatic tumor	1.3	1.0	.03	2.1	0.6	<.01
AIDS	0.9	1.0	.36	0.1	0.1	.60
Per capita medical expenditures^c^, $	28,350	16,417	<.01	10,857	2,315	<.01
**Connecticut**						
Sample, N	4,409	17,895	–	8,530	147,690	–
Mean age, y	53.3	43.7	<.01	46.1	36.8	<.01
Female, %	63.0	50.9	<.01	54.5	62.0	<.01
**Race/ethnicity, %**						
White	31.5	46.7	<.01	37.4	47.7	<.01
Black	22.1	22.9	.30	23.6	21.1	<.01
Hispanic	44.9	29.0	<.01	33.0	27.8	<.01
Other	1.5	1.4	.80	5.9	3.3	<.01
**Comorbidity^b^, %**						
Dementia	0.6	0.3	<.01	0.1	0.0	<.01
Chronic pulmonary disease	33.7	21.5	<.01	19.3	11.9	<.01
Rheumatic disease	2.4	2.1	.13	1.2	0.7	<.01
Peptic ulcer disease	1.3	0.7	<.01	0.9	0.4	<.01
Hemiplegia	0.9	1.0	.49	0.3	0.1	<.01
Any malignancy	6.9	4.7	<.01	4.0	1.7	<.01
Mild liver disease	4.0	2.2	<.01	1.7	0.4	<.01
Moderate/severe liver disease	1.9	0.8	<.01	0.8	0.2	<.01
Metastatic tumor	1.0	0.9	.82	0.4	0.2	<.01
AIDS	3.9	5.3	<.01	0.9	0.6	<.01
Per capita medical expenditures^c^, $	29,026	24,438	<.01	12,753	4,584	<.01
**Florida**						
Sample, N	12,990	57,345	–	2,370	60,318	–
Mean age, y	53.2	43.9	<.01	41.2	31.8	<.01
Female, %	62.4	51.5	<.01	72.5	71.1	.15
**Race/ethnicity, %**						
White	38.7	46.7	<.01	35.3	39.8	<.01
Black	31.8	31.5	.47	33.2	32.8	.73
Hispanic	28.4	20.5	<.01	30.3	26.3	<.01
Other	1.1	1.4	.01	1.2	1.1	.61
**Comorbidity^b^, %**						
Dementia	0.6	0.4	<.01	0.0	0.0	.44
Chronic pulmonary disease	35.2	20.2	<.01	17.7	8.0	<.01
Rheumatic disease	3.7	2.2	<.01	1.4	0.7	<.01
Peptic ulcer disease	2.2	1.0	<.01	1.2	0.4	<.01
Hemiplegia	1.2	1.3	.82	0.3	0.1	<.01
Any malignancy	12.4	6.7	<.01	6.8	1.6	<.01
Mild liver disease	3.0	1.5	<.01	0.8	0.1	<.01
Moderate/severe liver disease	1.2	0.6	<.01	0.2	0.0	<.01
Metastatic tumor	1.3	1.0	<.01	0.9	0.2	<.01
AIDS	5.3	7.7	<.01	1.7	1.7	.95
Per capita medical expenditures^c^, $	23,367	16,250	<.01	8,935	2,694	<.01
**Illinois**						
Sample, N	14,063	73,432	–	16,071	352,249	–
Mean age, y	53.2	43.7	<.01	44.2	35.2	<.01
Female, %	60.4	47.9	<.01	64.6	76.3	<.01
**Race/ethnicity, %**						
White	37.2	37.0	.60	41.8	52.7	<.01
Black	48.5	52.8	<.01	24.3	25.5	<.01
Hispanic	12.3	8.8	<.01	27.0	17.9	<.01
Other	2.0	1.3	<.01	7.0	3.9	<.01
**Comorbidity^b^, %**						
Dementia	0.5	0.2	<.01	0.1	0.0	<.01
Chronic pulmonary disease	38.3	21.7	<.01	19.0	8.9	<.01
Rheumatic disease	3.0	1.9	<.01	1.4	0.7	<.01
Peptic ulcer disease	2.8	1.5	<.01	1.9	0.8	<.01
Hemiplegia	1.7	1.4	.03	0.4	0.1	<.01
Any malignancy	13.2	7.5	<.01	9.5	4.4	<.01
Mild liver disease	2.6	1.3	<.01	0.8	0.2	<.01
Moderate/severe liver disease	1.1	0.5	<.01	0.3	0.1	<.01
Metastatic tumor	1.4	1.2	.03	0.6	0.2	<.01
AIDS	1.8	2.8	<.01	0.3	0.2	.08
Per capita medical expenditures^c^, $	21,328	11,100	<.01	8,085	2,077	<.01
**Iowa**						
Sample, N	2,769	13,879	–	935	40,994	–
Mean age, y	51.0	40.0	<.01	39.3	36.3	<.01
Female, %	67.3	53.2	<.01	71.0	67.6	.03
**Race/ethnicity, %**						
White	83.7	84.7	.18	74.5	81.7	<.01
Black	11.8	11.6	.80	13.6	10.7	<.01
Hispanic	2.7	1.8	<.01	8.1	4.2	<.01
Other	1.8	1.9	.91	3.7	3.3	.46
**Comorbidity^b^, %**						
Dementia	0.3	0.2	.28	0.0	0.0	.73
Chronic pulmonary disease	37.7	21.7	<.01	24.1	6.9	<.01
Rheumatic disease	2.3	1.4	<.01	1.6	0.3	<.01
Peptic ulcer disease	1.4	0.9	<.01	1.4	0.2	<.01
Hemiplegia	0.8	1.0	.30	0.3	0.1	<.01
Any malignancy	4.5	3.1	<.01	2.7	0.6	<.01
Mild liver disease	2.0	1.0	<.01	1.3	0.1	<.01
Moderate/severe liver disease	1.0	0.5	<.01	0.6	0.0	<.01
Metastatic tumor	0.8	0.8	.82	0.4	0.1	<.01
AIDS	0.3	0.3	.99	0.2	0.1	.48
Per capita medical expenditures^c^, $	25,248	18,649	<.01	11,912	2,318	<.01
**New York**						
Sample, N	5,111	36,073	–	1,200	25,951	–
Mean age, y	50.6	36.4	<.01	46.1	34.1	<.01
Female, %	57.3	43.6	<.01	46.2	55.4	<.01
**Race/ethnicity, %**						
White	51.3	57.6	<.01	51.5	58.2	<.01
Black	25.0	23.4	<.01	27.8	23.3	<.01
Hispanic	20.4	15.5	<.01	16.1	14.4	.09
Other	3.2	3.5	.34	4.7	4.1	.34
**Comorbidity^b^, %**						
Dementia	1.0	0.3	<.01	0.0	0.0	.52
Chronic pulmonary disease	32.0	13.2	<.01	21.3	9.6	<.01
Rheumatic disease	2.4	1.0	<.01	0.8	0.5	.18
Peptic ulcer disease	1.2	0.4	<.01	0.9	0.5	.03
Hemiplegia	2.1	1.5	<.01	0.6	0.1	<.01
Any malignancy	5.2	2.3	<.01	8.1	3.4	<.01
Mild liver disease	2.3	0.6	<.01	1.9	0.5	<.01
Moderate/severe liver disease	1.1	0.2	<.01	1.3	0.1	<.01
Metastatic tumor	0.9	0.4	<.01	1.7	0.6	<.01
AIDS	6.4	3.1	<.01	3.9	1.8	<.01
Per capita medical expenditures^c^, $	57,501	48,127	<.01	25,213	6,723	<.01
**Oklahoma**						
Sample, N	6,554	27,583	–	2,069	26,909	–
Mean age, y	51.6	43.1	<.01	44.0	35.1	<.01
Female, %	64.3	53.2	<.01	69.6	74.3	<.01
**Race/ethnicity, %**						
White	64.7	68.0	<.01	52.1	57.8	<.01
Black	16.7	18.3	<.01	11.5	11.8	.68
Hispanic	4.1	3.1	<.01	17.8	13.2	<.01
Other	14.5	10.5	<.01	18.6	17.2	.12
**Comorbidity^b^, %**						
Dementia	0.4	0.2	<.01	0.0	0.0	.36
Chronic pulmonary disease	37.4	24.7	<.01	19.9	13.8	<.01
Rheumatic disease	4.6	3.0	<.01	2.4	1.7	.03
Peptic ulcer disease	2.1	1.4	<.01	1.1	0.9	.35
Hemiplegia	1.9	1.4	<.01	0.7	0.1	<.01
Any malignancy	4.9	3.4	<.01	5.4	2.4	<.01
Mild liver disease	2.7	1.6	<.01	1.2	0.4	<.01
Moderate/severe liver disease	1.3	0.6	<.01	0.5	0.1	<.01
Metastatic tumor	0.9	0.9	.69	1.0	0.4	<.01
AIDS	0.3	0.7	<.01	0.3	0.4	.60
Per capita medical expenditures^c^, $	20,276	12,284	<.01	11,199	4,883	<.01

a *P* values are based on *t* test comparing means and χ^2^ test comparing proportions among people with and without diabetes.

b Comorbidities are adapted from Deyo et al (15).

c Unadjusted per capita mean annual medical expenditures (2012 US $).

### Outcome variables

We estimated medical expenditures in total and by component (inpatient care, outpatient care, prescription drugs). Inpatient expenditures included claims for hospital stays, but generally did not include physician and other provider services (11). Outpatient expenditures included all services not included in the inpatient or prescription drug claims, such as physician and other provider services, home health care, transportation, and outpatient facilities (11). Prescription drug expenditures included claims for prescription drugs, durable medical equipment, and vaccines (11). Total expenditure was the sum of these 3 individual components.

### Statistical analysis

For descriptive analyses, we calculated means for continuous variables and proportions for categorical variables, stratified by diabetes status. We tested the difference in means by using a *t* test and proportions by using a χ^2^ test between people with and without diabetes.

Not all enrollees had health care service use or medical expenditures during the year. In addition, expenditures were positively skewed for those who had expenditures. Therefore, we used a 2-part model to estimate inpatient care, outpatient care, and prescription drug expenditures by disability-based eligibility status (14). In the first part of the model, we used a generalized linear model with logit link and binomial distribution to estimate the probability of an individual having expenditures. In the second part, we used a generalized linear model with log link and gamma distribution to estimate medical expenditures.

In the models, we controlled for several covariates, including a dichotomously defined diabetes term (1 = diabetes diagnosis, 0 = without diabetes diagnosis). Choice of covariates was guided by past studies and data availability (8,12).The covariates included sex, age, age squared, race/ethnicity, and comorbidity indicators. Ten comorbidity indicators were included based on their prevalence (≥0.1%) and ICD-9-CM codes developed by Deyo et al (dementia, chronic pulmonary disease, rheumatic disease, peptic ulcer disease, hemiplegia, any malignancy, mild liver disease, moderate/severe liver disease, metastatic tumor, and AIDS) (15).

We predicted individual-level annual mean medical expenditures by diabetes status with parameter estimates from the 2-part model and values of the covariates. The mean medical expenditures associated with diabetes were calculated as the difference in the predicted per capita annual mean medical expenditures between enrollees with and without diabetes. We also calculated the cost ratios between enrollees with and without diabetes by dividing the predicted mean expenditures of enrollees with diabetes by those without diabetes. We estimated standard errors of predicted and excess medical expenditures associated with diabetes by using the nonparametric bootstrapping method with 1,000 replicates. All statistical analyses were conducted in 2017 by using SAS Enterprise Guide 7.1 (SAS Institute).

## Results

Table 1 reports characteristics and unadjusted total per capita medical expenditures for enrollees with and without diabetes by disability-based eligibility status. Among enrollees with disability-based eligibility, those with diabetes were more likely to be older, female, have larger proportions of diagnosed comorbidities, and have larger unadjusted total medical expenditures compared with those without diabetes. Characteristics of enrollees whose eligibility was not disability-based showed a similar pattern. The enrollees’ characteristics and unadjusted per capita total medical expenditures by disability and diabetes status displayed a similar pattern across the states.

Table 2 shows that, for disability-based enrollees, the estimated annual total excess medical expenditures associated with diabetes ranged from $6,183 (95% confidence interval [CI]: $5,627–$6,831) in Alabama to $15,319 (95% CI, $11,890–$19,023) in New York. Among enrollees with eligibility-based disability, those with diabetes spent 1.3 (New York) to 1.9 (California and Illinois) times more on total medical expenditures than those without diabetes (Table 2). In contrast, for nondisability enrollees, the estimated annual total excess medical expenditures associated with diabetes ranged from $4,985 (95% CI, $4,178–$5,912) in Alabama to $15,366 (95% CI, $12,100–$19,271) in New York (Table 2). The cost ratio ranged from 2.0 in Oklahoma to 3.3 in Iowa.

**Table 2:** Estimated Per Capita Mean and Excess Annual Total Medical Expenditures Among Adult Medicaid Enrollees Aged 19–64 Years by Disability-Based Eligibility and Diabetes Status, 8 States, 2012

With disability				Without disability			
With diabetes Mean (95% CI), $	Without diabetes Mean (95% CI), $	Excess^a^ (95% CI), $	Ratio^b^	With diabetes Mean (95% CI), $	Without diabetes Mean (95% CI), $	Excess^a^ (95% CI), $	Ratio^b^
**Alabama **							
13,900 (13,336–14,599)	7,717 (7,468–7,999)	6,183 (5,627–6,831)	1.8	9,530 (8,480–10,722)	4,545 (4,070–5,125)	4,985 (4,178–5,912)	2.1
**California**							
29,443 (27,969–31,112)	15,890 (15,169–16,708)	13,553 (12,061–15,288)	1.9	12,727 (11,586–14,071)	4,197 (3,804–4,641)	8,530 (7,535–9,645)	3.0
**Connecticut**							
30,818 (29,231–32,656)	19,631 (18,630–20,616)	11,187 (9,446–13,043)	1.6	14,901 (14,071–15,840)	6,202 (5,883–6,537)	8,699 (8,038–9,438)	2.4
**Florida**							
24,240 (23,471–25,274)	15,810 (15,365–16,310)	8,430 (7,610–9,381)	1.5	11,227 (10,201–12,617)	4,197 (3,837–4,581)	7,030 (6,188–8,192)	2.7
**Illinois**							
22,925 (21,963–24,009)	12,340 (11,887–12,805)	10,585 (9,675–11,690)	1.9	9,882 (9,269–10,615)	3,433 (3,207–3,699)	6,449 (5,966–7,060)	2.9
**Iowa**							
25,786 (24,166–27,784)	16,060 (15,230–17,000)	9,726 (8,179–11,641)	1.6	13,269 (11,584–15,570)	4,009 (3,431–4,601)	9,260 (7,895–11,282)	3.3
**New York**							
60,520 (57,377–64,000)	45,201 (43,487–46,912)	15,319 (11,890–19,023)	1.3	28,030 (24,147–32,363)	12,664 (10,867–14,612)	15,366 (12,100–19,271)	2.2
**Oklahoma**							
20,953 (19,910–22,193)	12,555 (12,024–13,130)	8,398 (7,359–9,619)	1.7	12,490 (11,267–14,058)	6,189 (5,637–6,796)	6,301 (5,246–7,632)	2.0

a Excess: difference between mean estimated expenditures of enrollees with and without diabetes. All excess expenditures are statistically significant (*P* < .001).

b Ratio: cost ratio of total medical expenditures of enrollees with diabetes to those without diabetes.

**Figure 1:**
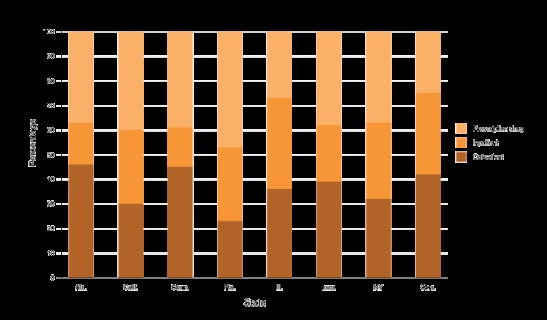
Percentage of inpatient care, outpatient care, and prescription drug expenditures of the total medical expenditures associated with diabetes among Medicaid adults with disability-based eligibility aged 19–64 years, 2012. **Table:** 

**Figure 2:**
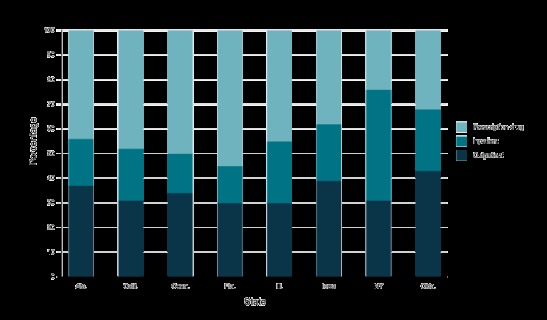
Percentage of inpatient care, outpatient care, and prescription drug expenditures of the total medical expenditures associated with diabetes among Medicaid adults without disability-based eligibility aged 19–64 years, 2012. **Table:** 

The composition of the excess total medical expenditures associated with diabetes is presented in Figure 1 for disability-based enrollees and Figure 2 for nondisability-based enrollees. Among disability-based enrollees in Alabama, Connecticut, Iowa, and Oklahoma, the largest share of expenditures was for outpatient care (46%, 45%, 39%, and 42%, respectively) (Figure 1). For California, Florida, and New York, the largest share was for prescription drugs (40%, 46%, and 37%, respectively). For Illinois, the largest share (37%) was for inpatient care. Among nondisability-based enrollees, for Alabama, California, Connecticut, Florida, and Illinois, prescription drugs accounted for the largest share (44%, 48%, 50%, 55%, and 45%, respectively) (Figure 2). For Iowa and Oklahoma, outpatient care had the largest share (39% and 43%, respectively). Finally, for New York, inpatient care had the largest share (45%).

State	Outpatient	Inpatient	Prescription Drug
Alabama	46	17	37
California	30	30	40
Connecticut	45	16	39
Florida	23	30	46
Illinois	36	37	28
Iowa	39	23	38
New York	32	31	37
Oklahoma	42	33	25

State	Outpatient	Inpatient	Prescription Drug
Alabama	37	19	44
California	31	21	48
Connecticut	34	16	50
Florida	30	15	55
Illinois	30	25	45
Iowa	39	23	38
New York	31	45	24
Oklahoma	43	25	32

## Discussion

A previous study showed that the financial burden imposed by diabetes on state Medicaid programs was substantial (7). Our study also supported this conclusion. Our estimated cost ratios comparing enrollees with diabetes to those without diabetes are also within the range of a past research review, which found that the cost for people with diabetes was 1.5 to 4.4 times more than for those without diabetes (16). However, we showed that the per capita medical expenditures associated with diabetes varied greatly across states. The highest-spending state studied (New York) had per capita medical expenditures 3 times more than the lowest-spending state (Alabama). These variations in diabetes-associated medical expenditures imply that using estimates from national level data to monitor the financial burden of diabetes and inform Medicaid policy decision at the state level could be misleading.

Factors that contribute to the state variations in excess diabetes expenditures are complex, and identifying those reasons is beyond the scope of this study. However, we did decompose the total expenditure into service volume and payment per service by component (data not shown) to see how each of the 2 factors has contributed to the total excess expenditure. In general, high-expenditure states (California, Connecticut, and New York) tended to have higher payment per unit of service than low-expenditure states (Alabama, Florida, Illinois, Iowa, and Oklahoma), in particular for prescription drug fills. For example, for disability-based enrollees with diabetes, high-expenditure states paid 1.2 to 2.4 times more per prescription drug fill, on average, than low-expenditure states. However, it is less clear if high-expenditure states had a higher service volume per enrollee than low-expenditure states. The variation in medical expenditures could be explained in part by differences in eligibility criteria, benefits, and policies of state Medicaid programs (10). Other factors such as availability of state funding for public programs, access to care, availability of health services, and demand for services could have contributed to the variation (10).

Whether or not higher medical spending associated with diabetes leads to better diabetes care outcomes remains unclear, and is also beyond the scope of our study. Previous studies on this question showed that health care spending and the health status of people living in the state were not directly correlated (17). Other factors, such as economic stability, neighborhood environment, education, and healthcare systems (18), along with social services and public health spending (19), may play a more important role than per capita spending in determining overall health outcomes.

Our findings showed that medical expenditures associated with diabetes also varied by disability-based eligibility status. Those with disability-based eligibility had much higher absolute values of medical expenditure than those without disability-based eligibility (ranging from 1.4 to 2.3 times greater). In general, individuals with disability-based eligibility have a higher prevalence of chronic conditions, and therefore have higher medical expenditures than those without disability-based eligibility (20). In contrast, the cost ratios (between enrollees with diabetes and those without diabetes) among studied states for those with disability-based eligibility were much smaller (1.3–1.9) than those without disability-based eligibility (2.0–3.3). A similar pattern was observed by Shrestha and colleagues among youth by using MarketScan Medicaid data from 2008–2012. They showed that per capita annual diabetes-related medical expenditures were larger among youth with disability than those without disability ($9,944 vs $9,046), but the cost ratio for those with disability was much lower (1.7 vs 3.5) (21). This result may stem from the fact that individuals with disability-based eligibility already have numerous chronic conditions and incur high medical expenditures, so that having an additional condition (diabetes, in this case) creates less incremental financial burden than it does for those whose eligibility is not disability-based (22). The variation in diabetes-associated medical expenditures by eligibility criteria implies that medical needs differ for those with and without disability, requiring diverse interventions and strategies from stakeholders.

In addition, for disability-based enrollees, measured by cost ratios, diabetes imposed the largest burden in California and Illinois. But using absolute dollar amount as the measure, diabetes imposed the largest burden in New York (Table 2). For nondisability-based enrollees, Iowa had the largest diabetes burden if cost ratio were used and New York had the largest burden if absolute dollar amount were used (Table 2). Thus, both absolute and relative differences in estimated medical expenditures between those diagnosed with diabetes and without diabetes are important measures to better understand the financial burden of diabetes in the Medicaid population.

Our results also showed that the proportion of components in total medical expenditures differed by state and eligibility criteria. This could be explained in part by the differences in coverage benefits, reimbursements, and cost-sharing policies of state Medicaid programs (23,24). Among 4 states (Alabama, Connecticut, Iowa, and Oklahoma) that had average physician fees greater than the Medicaid national average, outpatient care expenditures accounted for a larger share of excess total medical expenditures (data not shown).

For inpatient care policies, New York was the only studied state that required no prior approval for inpatient hospital services, imposed no limit on number of service days, and had higher than national average payment per inpatient hospital stay. Historically, New York has tended to offer many optional services and paid much higher hospital fees than many other states (25), which might explain why its inpatient expenditures accounted for a higher share of total diabetes-related medical expenditures.

As for prescription drug policies, such as prior authorization requirement, copayment, and monthly limit on prescription drugs, no clear picture emerged to explain the variation of composition in total diabetes excess expenditures. Prior research suggests the reason for the largest share of prescription drug expenditures could be due mainly to higher volume and prescription drug prices (newer and more expensive drugs) used to treat diabetes or diabetes-related complications (or due to health behavior needs) (8). In addition, more and better use of disease management services at outpatient or primary care settings could have resulted in greater prescription utilization and adherence (26).

Studies have shown that type 2 diabetes is preventable through prevention efforts such as the National Diabetes Prevention Program, a structured lifestyle change program, and could potentially reduce the financial burdens of state Medicaid programs (27). This program has been found to reduce the risk of type 2 diabetes by more than half and to be cost-effective (28). For those with diabetes, disease management is critical to prevent diabetes-related complications. Diabetes self-management education and support (DSMES) provides support for informed decision-making and self-care practices, and could improve health outcomes and reduce the financial burdens of state Medicaid programs (29). DSMES has been shown to reduce risk of diabetes complications and to be cost-effective (30).

### Limitations

Our study has several limitations. First, we focused on FFS enrollees; therefore, the results may not apply to the Medicaid population enrolled in other insurance programs. Second, our findings may not be applicable to other states, owing to the heterogeneity of state Medicaid programs and population characteristics. Third, medical expenditures for type 1 and type 2 diabetes are different, but limitations of coding from administrative claims data and the nature of the treatment schemes prevented us from reliably distinguishing them. However, because most adults have type 2 diabetes, these results likely primarily reflect medical expenditures associated with type 2 diabetes than type1 diabetes. Fourth, the cost of a chronic condition that was estimated by using claims data may depend on the specific algorithm used for identifying people with that condition. Thus, our estimated cost of diabetes might differ if we used another algorithm to identify those with diabetes. Finally, no out-of-pocket costs or indirect costs were available, which would have provided a broader perspective of financial burden associated with diagnosed diabetes.

Because states have options in setting Medicaid policies, variations in eligibility criteria and specific benefit coverage across states could result in different diabetes-associated medical expenditures. Past studies on these expenditures in the Medicaid population mainly focused on national-level estimates. State-specific estimates were either not available or were extrapolated from national data. Our estimates for state-level diabetes-associated medical expenditures can be used by the 8 state Medicaid programs to prepare healthcare resources needed for diabetes care and to assess the financial benefits of diabetes prevention and management programs.
